# From ideal to practical: Heterogeneity of student-generated variant lists highlights hidden reproducibility gaps

**DOI:** 10.1371/journal.pcbi.1013552

**Published:** 2025-10-16

**Authors:** Rumeysa Aslıhan Ertürk, Abdullah Asım Emül, Büşra Nur Darendeli-Kiraz, Fatma Zehra Sarı, Mehmet Arif Ergün, Mehmet Baysan

**Affiliations:** 1 Computer Engineering Department, Istanbul Technical University, Istanbul, Türkiye; 2 Bioengineering Department, Yıldız Technical University, Istanbul, Türkiye; 3 Türkiye Health Data Research and Artificial Intelligence Applications Institute, Health Institutes of Türkiye, Istanbul, Türkiye; 4 Izmir Biomedicine and Genome Center, Dokuz Eylul University, Izmir, Türkiye; Montreal, CANADA

## Abstract

Next-generation sequencing (NGS) technologies offer detailed and inexpensive identification of the genetic structure of living organisms. The massive data volume necessitates the utilization of advanced computational resources for analyses. However, the rapid accumulation of data and the urgent need for analysis tools have caused the development of imperfect software solutions. Given their immense potential in clinical applications and the recent reproducibility crisis discussions in science and technology, these tools must be thoroughly examined. Typically, NGS data analysis tools are benchmarked under homogeneous conditions, with well-trained personnel and ideal hardware and data environments. However, in the real world, these analyses are done under heterogeneous conditions in terms of computing environments and experience levels. This difference is mostly overlooked, therefore studies that examine NGS workflows generated under various conditions would be highly valuable. Moreover, a detailed assessment of the difficulties faced by the trainees would allow for improved educational programs for better NGS analysis training. Considering these needs, we designed an elective undergraduate bioinformatics course project for computer engineering students at Istanbul Technical University. Students were tasked to perform and compare 12 different somatic variant calling pipelines on the recently published SEQC2 dataset. Upon examining the results, we have realized that despite seeming correct, the final variant lists created by different student groups display a high level of heterogeneity. Notably, the operating systems and installation methods were the most influential factors in variant-calling performance. Here, we present detailed evaluations of our case study and provide insights for better bioinformatics training.

## Introduction

Science is an inherently cumulative endeavor, where each study builds on past research. The reliability of this progression depends crucially on the repeatability of scientific results, ensuring that subsequent research is based on solid and verifiable foundations. Reproducibility is, therefore, fundamental to the integrity and credibility of scientific inquiry. It validates the accuracy of findings and allows for the consistent development of new theories and applications. Nevertheless, the scientific community has increasingly recognized the phenomenon of a “reproducibility crisis” [1]. This refers to the growing concern that a significant proportion of published research cannot be reliably reproduced by independent researchers, casting doubt on the validity of many scientific findings. Many studies show that reproducibility rates are particularly low in certain fields [2,3] such as social, behavioral [4–7], and life sciences [8–11]. For example, in their study, Errington et al. initiated the “Reproducibility Project” in cancer biology, aiming to systematically evaluate the reproducibility of significant findings in the field [12]. Through an extensive investigation of 23 papers published in high-impact journals, they found that only 46% of the reported results could be successfully replicated [13].

Next-generation sequencing (NGS) refers to high-throughput methods that have significantly reduced the time and cost of sequencing. These technologies allow for the study of entire genomes and are widely used in genomics research [14]. NGS technologies are applicable in many clinical areas, such as disease diagnosis, prognosis prediction, and treatment optimization. Moreover, detailed profiling of the genome makes NGS technologies a key tool in personalized medicine [15]. Given its direct impact on human health, bioinformatics requires a closer examination of reproducibility [16], particularly in terms of clinical genomic data analyses. NGS has significantly accelerated data generation in genomics [17]. This growth in data has driven the need to develop new sophisticated computational tools and analysis pipelines. Although many researchers contribute valuable tools to the field, most of these tools do not meet comprehensive software engineering standards. Detailed documentation and extensive reliability and stability tests are major requirements for developing high-quality software. However, these aspects are often neglected due to the time-intensive nature of their implementation. The urgent need for NGS analysis tools has been a primary driver behind this oversight. Unfortunately, the lack of thorough testing and documentation significantly undermines the reproducibility of results and compromises the overall quality and reliability of NGS analyses.

There is a growing demand for bioinformatics experts, given the critical role of NGS research in clinical applications. Integrating NGS analysis into university education programs is an important step to meet this demand. The inclusion of NGS projects in computer science and engineering programs would especially help to improve the quality of NGS-oriented software development. Moreover, training and reproducibility assessment can be combined by constructing course projects that focus on the reproduction of published NGS analysis. Few studies in the bioinformatics literature used this idea for varying problems. Karathanasis et al. developed a graduate-level course that included a term project where students reproduced published NGS analysis articles. Their goal was to expose students to real-world data analysis challenges and contribute to discussions on analytical reproducibility. In their study, four groups (seven students) managed to confirm 39% of the figures, producing results consistent with the original findings [18]. Similarly, Cokelear et al. reported on a three-year project in which 123 graduate students attempted to reproduce one of the two selected RNA-sequencing studies. They found significant variability in the results submitted by the students. The differences arose from data acquisition, differences in statistical analyses, and unreported details in the original studies, such as tool versions, algorithm parameters, and p-value thresholds [19].

Given the critical role of NGS research in clinical applications, a thorough analysis of reproducibility in NGS workflows remains urgently needed. Although some studies have addressed this issue [20,21], the literature still lacks comprehensive works that explore the various dimensions of reproducibility in NGS analyses. It is essential to emphasize that the primary focus of this study is not on the reproducibility of the algorithms themselves, but rather on the computational reproducibility within the context of real-world NGS analyses. Such analyses are rarely conducted under ideal conditions. In practice, they are performed by individuals with diverse backgrounds, using different computational environments and hardware setups. As a result, it is important to assess how these variations in computational contexts may influence the reproducibility of the outcomes. The reproducibility examined in this study is based on these non-ideal, practical conditions. Additionally, there is a pressing need for initiatives aimed at training bioinformatics researchers who possess strong foundations in both computational and life sciences. To address these issues, we developed an undergraduate course for computer engineering students. We aimed to evaluate the reproducibility of current tools for somatic variant calling and to introduce bioinformatics concepts to computer engineering students. This elective course was offered in the Computer Engineering Department at Istanbul Technical University. Throughout the course, students received both theoretical and practical instruction on genomics, variant calling, and data science. The course also included a semester-long project focused on somatic variant calling.

We chose somatic variant calling because somatic sequencing is crucial for identifying tumor-initiating mutations, and it presents significant challenges for analysis. Unlike inherited variants, the heterogeneity of mutations and copy number changes make somatic sequencing particularly difficult. Due to these challenges, the concordance rate among variant callers is low [22]. Recently, the FDA-led SEQC2 project concluded. One of its major outcomes is a thoroughly analyzed and characterized benchmark sample and dataset to assess the performance of somatic sequencing pipelines [23]. This dataset includes comprehensive sequencing of normal and tumor cell lines from the same individual. After analyzing these samples, researchers also provided a list of high-confidence variants for high-confidence regions to be used in performance evaluations of alternative pipelines.

In our course, we used the SEQC2 dataset and tasked students with identifying somatic variants by comparing tumor and normal samples. Students were required to construct and run 12 pipelines (using two different aligners, three different variant callers, and performing or skipping base recalibration) on a single tumor-normal pair. The final project presentation and report included statistical analyses to evaluate pipelines’ performances using the declared high-confidence variants. Students also created detailed visualizations, including heatmaps and principal component analysis (PCA) diagrams, to present the concordance and accuracy of the alternative pipelines. Additionally, we prepared a questionnaire to measure the time and effort required for each step of the analysis. After collecting the 12 resulting variant call format (VCF) files and questionnaire responses of each of the 11 groups, we conducted a comprehensive study to identify factors that affected variant calling performance.

We compared 132 student-submitted VCF files (11 groups × 12 pipelines) with the high-confidence variant list from the SEQC2 consortium. We evaluated the performance of each pipeline in terms of precision, recall, and F1-score. We also examined the impact of several factors on these metrics, such as operating environment, installation method, and time spent on different phases of the project. To the best of our knowledge, this study introduces several aspects that have been largely overlooked in the literature. First, no prior research has specifically examined the findings of undergraduate students in computer science-related fields. Second, the incorporation of a survey capturing hardware and software specifications of the machines used adds a novel dimension. Third, this study uniquely explores the impact of time allocation across different phases of the project, distinguishing it from existing research.

The remainder of this paper is organized into three sections. First, we briefly describe the course project and the methods used for the analyses. In the second section, we present the results of our analyses of student VCF files and questionnaire responses. Lastly, we discuss and interpret our findings.

## Materials and methods

### Project description

The project was designed to provide practical experience to the students regarding NGS analysis algorithms. The project required students to perform somatic variant calling on FASTQ files via COSAP (Comparative Sequencing Analysis Platform) [24]. COSAP enables users to create and compare NGS pipelines, which is crucial to assessing and establishing reproducibility, especially for difficult problems such as somatic sequencing analysis.

**Fig 1:**
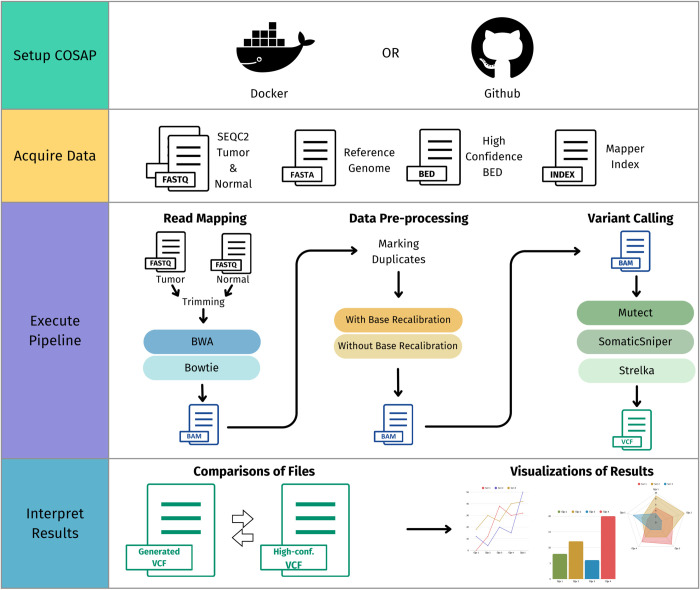
Steps of the pipelines. Each pipeline has trimming and duplicate marking steps. The rest of the steps have alternative options. Each group created 12 VCF documents (2 aligners, 2 options for base recalibration, three variant calling algorithms).

The primary objective of the project was to run somatic sequencing analyses and evaluate the effectiveness of different NGS tools by creating multiple pipelines for detecting single-nucleotide polymorphisms (SNPs). The students were tasked with creating 12 pipelines with different configurations. For each pipeline, FASTQ files for tumor and normal samples were to be trimmed, aligned, and duplicate marking was to be performed. The 12 different pipelines were constructed through the combinations of the following parameters: (a) 2 base- recalibration options (applying and not applying), (b) 2 mapping algorithms (BWA [25], and Bowtie2 [26]), (c) 3 variant calling algorithms (Mutect [27], SomaticSniper [28], Strelka [29]). Fig 1 depicts an overview of the analysis pipeline along with possible parameters.

Further details related to the project description are shared in the S1 Text.

### Project execution

The project requirements were communicated to the students through a detailed project description document. This document provided step-by-step instructions for downloading the necessary data, installing the required tools, and suggestions for the final analyses. Throughout the semester, we maintained continuous interaction with the students, observing that different groups encountered differing challenges at various stages of the variant calling process. We noted that groups reported varying execution times for different stages, leading us to hypothesize a potential relationship between the time invested in each stage and the resultant variant calling performance.

Project presentations, reports, and VCF files were submitted through the university’s e-learning system. After completing the grading process, we shared a questionnaire to gather feedback on the students’ experiences. The questionnaire (Table A in S1 Tables) included both quantitative and qualitative questions about different phases of the project. We specifically asked students about the system they used (hardware, operating environment etc.) and the time they spent on various stages. We evaluated the relationship between these factors and the variant calling performance.

## Results

### Preliminary data analysis

Firstly, we compared the variant counts across different pipeline configurations to assess the similarity of the results obtained by the students. The high-confidence variant list from the SEQC2 consortium, which includes 1161 SNPs after applying exome filtering, serves as the benchmark for this comparison [30]. Instead of a single pipeline, SEQC2 employed an ensemble approach that merged results from multiple algorithms and alignment methods (e.g., MuTect2, Strelka2; BWA-MEM) and integrated evidence across many independent sequencing replicates of the same tumor–normal samples generated at multiple centers and platforms (HiSeq, NovaSeq). By prioritizing variants consistently supported across tools and replicates—and resolving disagreements with orthogonal evidence (e.g., long reads)—they distilled a robust, high-confidence reference set.

The results demonstrate significant variability in the number of variants detected and the rate of similarity to the high-confidence variant list, as seen in Table 1. For instance, the SomaticSniper-NB-BWA (SomaticSniper as the variant caller, no base recalibration, and BWA as the aligner) configuration shows remarkable consistency across many groups but reports more variants than expected. On the other hand, pipelines using Mutect exhibit more consistent outcomes among groups, closely aligning with the expected high-confidence variant count.

**Table 1:** Variant counts by group and pipeline configuration.

Pipeline	G1	G2	G3	G4	G5	G6	G7	G8	G9	G10	G11	HC
**MT-NB-BWA**	926 (52.7%)	715 (44.4%)	518 (22.4%)	4103 (21.5%)	771 (50.4%)	745 (45.3%)	1078 (67.8%)	745 (45.3%)	275 (19.5%)	435 (18.8%)	235 (12.9%)	1161
**MT-NB-BOW**	880 (56.0%)	892 (64.0%)	668 (32.9%)	3566 (23.8%)	385 (28.9%)	485 (33.4%)	952 (68.5%)	485 (33.4%)	233 (17.6%)	1819 (9.4%)	381 (20.9%)	
**MT-YB-BWA**	1052 (69.4%)	1044 (69.4%)	851 (36.8%)	2735 (24.6%)	838 (55.2%)	663 (42.8%)	1050 (69.4%)	663 (42.8%)	322 (21.4%)	489 (22.4%)	144 (8.4%)	
**MT-YB-BOW**	637 (45.1%)	588 (44.3%)	734 (37.3%)	2836 (29.6%)	939 (69.6%)	477 (34.0%)	1004 (68.0%)	477 (34.0%)	324 (23.6%)	1830 (15.6%)	246 (14.3%)	
**SS-NB-BWA**	2889 (27.4%)	2889 (27.4%)	9397 (9.0%)	2889 (27.4%)	2889 (27.4%)	2889 (27.4%)	2889 (27.4%)	2889 (27.4%)	2889 (27.4%)	8480 (8.5%)	8480 (8.5%)	
**SS-NB-BOW**	2406 (29.1%)	2406 (29.1%)	6858 (11.1%)	2406 (29.1%)	2406 (29.1%)	2406 (29.1%)	2406 (29.1%)	2406 (29.1%)	2406 (29.1%)	6137 (10.5%)	6858 (11.1%)	
**SS-YB-BWA**	2313 (32.6%)	2313 (32.6%)	8290 (9.9%)	1890 (30.5%)	2313 (32.6%)	2313 (32.6%)	2313 (32.6%)	2312 (32.6%)	2312 (32.6%)	7492 (9.4%)	7492 (9.4%)	
**SS-YB-BOW**	1752 (36.6%)	1752 (36.6%)	5521 (13.2%)	1752 (36.6%)	1752 (36.6%)	1752 (36.6%)	1752 (36.6%)	1752 (36.6%)	1752 (36.6%)	4934 (12.4%)	5521 (13.2%)	
**ST-NB-BWA**	2204 (38.9%)	2204 (38.9%)	3169 (27.8%)	108864 (0.9%)	2204 (38.9%)	2204 (38.9%)	2203 (39.0%)	2201 (39.0%)	2201 (39.0%)	2832 (25.8%)	2898 (25.3%)	
**ST-NB-BOW**	1313 (50.6%)	1313 (50.6%)	1786 (39.3%)	90416 (1.1%)	1313 (50.6%)	1313 (50.6%)	1313 (50.6%)	1313 (50.6%)	1313 (50.6%)	107672 (0.8%)	1839 (38.3%)	
**ST-YB-BWA**	1927 (43.5%)	1927 (43.5%)	2809 (30.9%)	83288 (1.2%)	1927 (43.5%)	1926 (43.5%)	1926 (43.5%)	1924 (43.6%)	1924 (43.6%)	2508 (28.5%)	2575 (27.9%)	
**ST-YB-BOW**	1173 (54.8%)	1173 (54.8%)	1549 (43.8%)	65208 (1.5%)	1173 (54.8%)	1173 (54.8%)	1173 (54.8%)	1173 (54.8%)	1173 (54.8%)	81825 (1.1%)	1605 (42.6%)	

The table shares the number of variants found (upper number) and the rate of Jaccard similarity (intersection-over-union) to the high confidence variant list (lower number in brackets) per group and pipeline configuration. The final column presents the variant count of the high-confidence variant file. The cell color represents the rate of similarity to the high-confidence variant list, with darker shades of green indicating a closer result. The abbreviations ‘MT’, ‘SS’, and ‘ST’ denote Mutect, SomaticSniper, and Strelka, while ‘YB’ and ‘NB’ signify the presence and absence of base recalibration, respectively. ‘HC’ stands for high-confidence and stores the number of variants in the high-confidence variant list.

This variability highlights a critical issue with reproducibility in variant calling. Major discrepancies were observed in variant counts across different pipeline configurations. This highlights the need for a thorough examination of the factors contributing to these inconsistencies.

**Fig 2:**
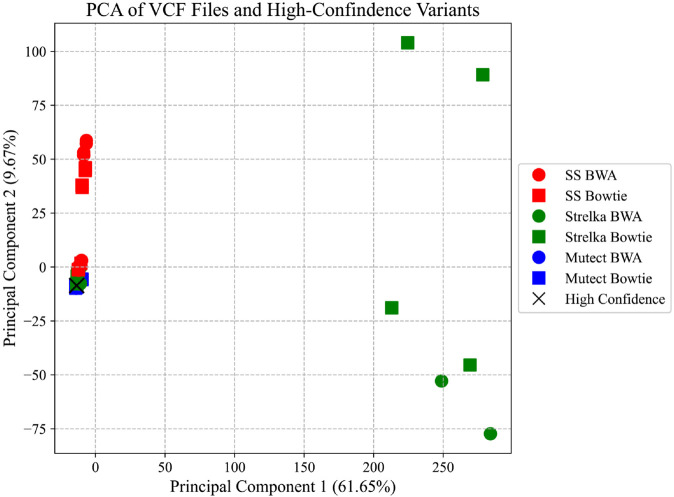
PCA analysis results. PCA for each pipeline configuration along with the high-confidence variant list, grouped by the aligner and variant calling algorithm. ‘SS’ stands for SomaticSniper variant caller algorithm.

Principal Component Analysis (PCA) was performed to assess the agreement between different pipelines using various mapping and variant calling algorithms. The analysis was based on whether each variant appeared in at least one of the students’ VCF files or in the high-confidence variant list. A binary matrix was created from 133 VCF files (132 from students and 1 from a high-confidence source) based on the presence or absence of specific variants. This binary matrix was then analyzed using PCA, with the results displayed in Fig 2. This figure reveals that variant lists generated using the Mutect variant caller show a high degree of similarity with the high-confidence variant list, irrespective of the mapping algorithm employed. On the other hand, while some variant lists produced using SomaticSniper exhibited high similarity with high-confidence variants, others diverged significantly. Finally, some variant lists derived using Strelka displayed significant divergence from the high-confidence variant list, indicating a weaker overall performance. These findings are consistent with previous observations based on variant counts and similarity metrics. Fig A in S1 Figs illustrates that these divergent variant lists were obtained using WSL as the operating environment and Docker as the installation method.

### General performance evaluation

**Fig 3:**
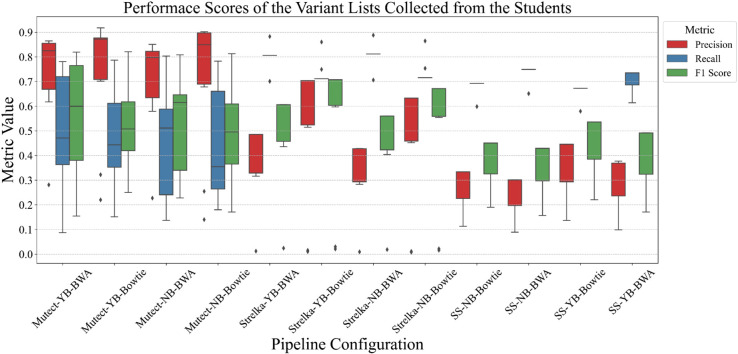
Performance scores of variant lists collected from the students It shows the distribution of scores for each pipeline combination for 132 VCF files. ‘YB’ and ‘NB’ denote applying and not applying base recalibration, respectively. ‘SS’ is the abbreviation of the SomaticSniper variant caller algorithm.

VCF files collected from students were benchmarked against a high-confidence variant list provided by the SEQC2 consortium [23]. Performance metrics, including precision, recall, and F1-score, were used for evaluation (Table B in S1 Tables for formulae and Tables C-E for the exact values). Fig 3 illustrates the distribution of these performance metrics across 12 different pipelines.

We observed significant variability in performance among the groups. Pipelines with Mutect exhibit relatively high precision, though they display considerable variability in recall with an average of around 0.5. Conversely, pipelines employing Strelka and SomaticSniper consistently demonstrate lower precision than recall. There are many VCF files with significantly low precision scores in pipelines using Strelka. This suggests a lack of robustness in Strelka under varying conditions.

Furthermore, when other parameters are kept constant, the influence of the mapping algorithm is most noticeable in pipelines using Strelka. For other variant callers, the impact of the aligner on performance is minimal. Contrary to expectations, base recalibration does not significantly affect performance across pipeline configurations.

### Effects of different environments

**Fig 4:**
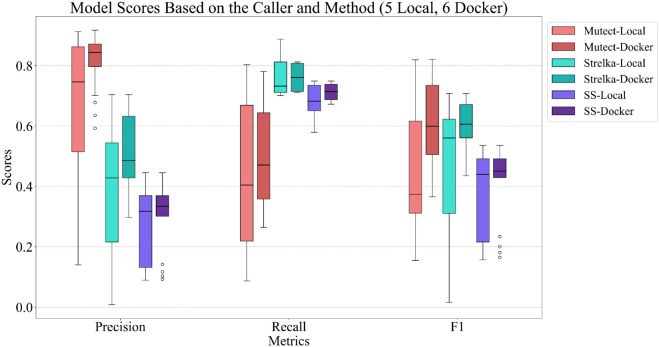
Performance comparison of variant callers using local installation versus Docker container. The box plots display the distribution of precision, recall, and F1-scores for different variant callers (Mutect, Strelka, and SomaticSniper) categorized by installation method (local vs. Docker).

As previously mentioned, students were given two options for setting up their computational environments: using a Docker image or manually installing the required packages. Six groups chose the Docker container method, while five groups opted for manual installation (Fig B in S1 Figs). Contrary to initial expectations, significant differences were observed in the variants identified using distinct methods. Fig 4 illustrates the distribution of performance metrics, including precision, recall, and F1-score, categorized by the variant caller algorithm to assess the impact of the installation method on algorithm performance. Two primary conclusions can be drawn from these metrics. Firstly, the use of Docker containers consistently enhances the average performance across all variant callers. Secondly, the lower variance observed with Docker suggests that Docker-based setups result in more robust and reliable pipelines (see Fig C in S1 Figs for the same effect with different aligner algorithms).

The choice of operating environment, like the installation method, is an important factor in the performance of the variant calling process. In this class, we allowed students to choose the operating environment they preferred to use, but we recommended using Linux. Five groups used Windows Subsystem for Linux (WSL). WSL is a feature introduced in Windows that allows users to run a full Linux environment directly on their Windows machine without needing to maintain separate virtual machines or dual-boot setups [31]. The remaining six groups used Linux natively.

**Fig 5:**
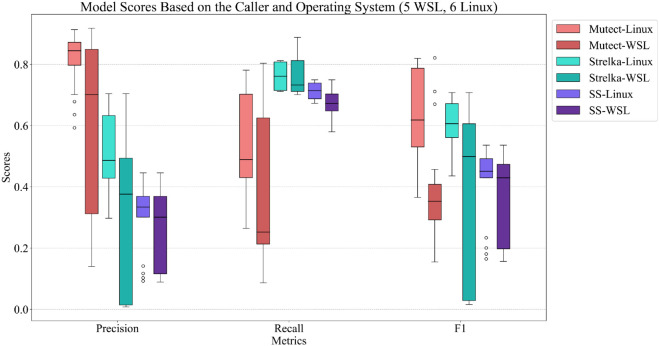
Performance comparison of variant callers using two operating environments: Linux and Windows Subsystem for Linux (WSL). The box plots display the distribution of precision, recall, and F1-score for different variant callers—Mutect, Strelka, and SomaticSniper—categorized by the operating environments (Linux vs. WSL).

Fig 5 compares different variant caller algorithms’ performance measures between operating environments in terms of precision, recall, and F1-score. The data makes it clear that using a native Linux environment gives a higher average performance for each variant caller when compared to using WSL. Moreover, WSL-based analysis displayed a higher variance of performance measures, indicating poor stability.

In particular, the F1-score for the Mutect algorithm is substantially higher when run natively. Similarly, the performances of the Strelka and SomaticSniper algorithms exhibit significant sensitivity to the operating environment, with notable variations among groups using WSL. These results underscore that employing a native Linux environment yields more consistent outcomes for these algorithms.

In summary, while WSL offers a convenient alternative for running Linux environments on Windows machines, native Linux installations provide superior and more reliable performance for variant calling tasks. This supports the recommendation to use Linux for such computationally intensive bioinformatics processes (see Fig D in S1 Figs for the same effect with different aligner algorithms).

### Factors that affect f1-scores

#### Time allocation.

Throughout the semester, we maintained continuous interaction with the students, observing that different groups encountered differing challenges at various stages of the variant calling process. We noted that groups reported varying execution times for different stages (Figs E and F in S1 Figs), leading us to hypothesize a potential relationship between the time invested in each stage and the resultant variant calling performance. Consequently, we requested students to estimate the time spent on each stage, categorizing it into five intervals: less than 2 hours, 2-4 hours, 4-8 hours, 8-16 hours, and more than 16 hours. The stages were divided into five steps: data downloading, mapping, variant calling, filtering, and analysis.

**Fig 6:**
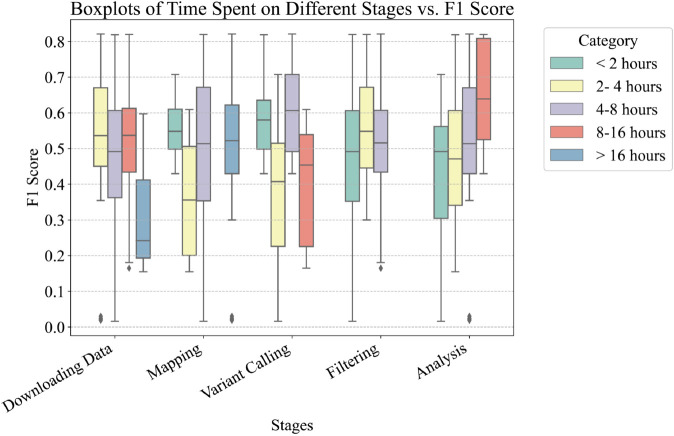
Box plots of time spent on different stages of the variant calling process versus the corresponding F1-score. The stages analyzed include downloading data, mapping, variant calling, filtering, and analysis.

As illustrated in Fig 6, the time allocated to each stage significantly influences the final variant calling performance. For instance, an increase in the time spent downloading FASTQ files correlates with a substantial decrease in average performance. This phenomenon could be attributed to the downloading of incomplete or faulty FASTQ files, as students reported connection failures while retrieving data from the European Nucleotide Archive (ENA). ENA provides a comprehensive database for the storage and access of nucleotide sequence data [32]. This observed correlation highlights the need to monitor the data downloading process to ensure completeness and integrity.

Similarly, spending a prolonged time on mapping and variant calling tends to reduce the performance of the results. This might be due to the algorithms’ inadequate error messages, which may terminate without flagging critical issues, resulting in incorrect outcomes. Conversely, an extended duration dedicated to the final analysis step, where students interpret their results by creating figures, correlates with improved performance. This suggests that students who invested considerable time into analysis were generally more successful in the preceding steps.

In conclusion, the analysis indicates that careful monitoring and efficient management of each stage, particularly data downloading and processing steps, are crucial for achieving high-performance variant calling results. Further investigation into optimizing these processes could yield more consistent and accurate outcomes.

#### External factors.

Previous analyses have demonstrated that several factors, including the operating environment, method of installation (whether local or using Docker), variant callers, and aligners, significantly influence the performance of the resulting F1-scores in variant calling tasks. To further investigate these relationships, a comprehensive Analysis of Variance (ANOVA) was performed [33]. This statistical method allows for the comparison of means across multiple groups and helps to identify whether any of the independent variables have a statistically significant effect on the dependent variable. Our dependent variable was the F1-score.

**Fig 7:**
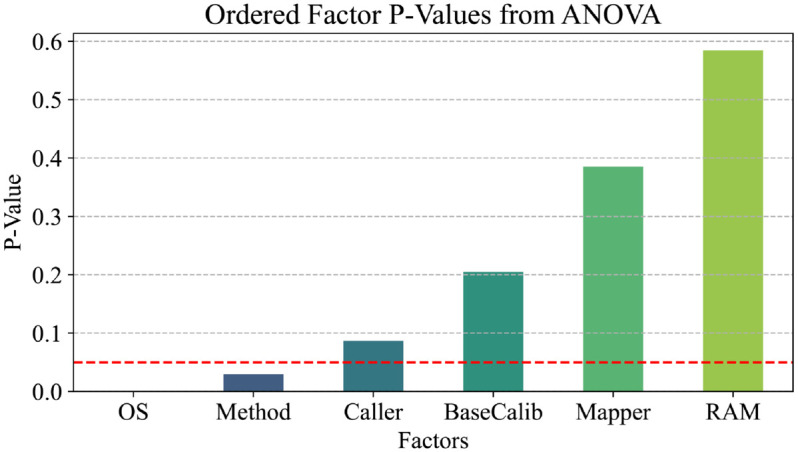
P-values obtained from ANOVA. A value lower than 0.05 indicates a statistically significant effect on the outcome.

Fig 7 presents the p-values corresponding to each factor analyzed in this study (see Table F in S1 Tables for the list of factors analyzed). Low p-values (typically less than 0.05) indicate a significant effect on the dependent variable, suggesting that the factor in question likely influences the results in a meaningful way. The results from the ANOVA analysis reveal that both the operating environment, specifically Linux versus WSL, and the installation method, whether local installation or deployment through Docker, exhibit statistically significant effects on the performance metrics of variant calling.

Additionally, while factors such as the choice of variant caller and aligner were also considered, they demonstrated less influence, as indicated by higher p-values in the analysis. This suggests that while they may contribute to the overall performance, their impact is not as pronounced as that of the operating environment and installation method. Overall, these findings underscore the importance of selecting appropriate configurations in computational workflows for NGS analysis, as the choice of operating environment and installation method can lead to substantial differences in performance outcomes.

## Discussion

Establishing solid reproducibility standards and training specialized personnel are two major components of the successful deployment of advanced technology. To address both of these issues in the context of NGS, we designed a course to introduce undergraduate computer engineering students to NGS analysis. The course project utilized a recent comprehensive benchmark dataset, which includes high-confidence variants for somatic sequencing. Somatic sequencing is critical for identifying tumor-initiating mutations, but it presents significant challenges. Unlike inherited variants, somatic mutations exhibit heterogeneity and copy number variations, making analysis particularly difficult. Consequently, concordance among different variant callers tends to be low. Given the challenges and importance of somatic sequencing, it serves as an ideal focus for reproducibility analyses.

Despite these challenges, most student groups in our course successfully completed the project. However, despite using the identical library versions and function parameters (Table G in S1 Tables), the results revealed a diverse range of errors that supported our previous findings regarding the poor standardization of analysis tools. The unexpected degree of discordant results, even with high-quality data, raises significant concerns about the reliability of somatic sequencing, especially in comparison to the more standardized approaches used in germline analyses.

There is an important distinction between computer-based and non-computer-based tools. For computational tools, it is often difficult to detect imperfect results. When an algorithm produces an output without any warnings or errors, many inexperienced users tend to accept the results without conducting thorough correctness testing. This oversight significantly affects computational analysis and poses a major threat to reproducibility.

Moreover, the pressure on developers to produce efficient, multifunctional tools for publication can compromise the rigorous testing necessary for reliable tool deployment. This tendency reinforces the need for strong software engineering principles within university curricula to ensure that students are equipped with the skills to develop robust and standardized analysis tools.

The challenge of merging advanced computational skills with genomic knowledge often leads to inadequately standardized tools that lack proper documentation and testing. Our course initially focused on three key variables: aligner, base recalibration, and variant caller, with plans to expand this scope in future iterations. By tailoring the course for computer engineering students, we aimed to empower them to effectively navigate the computational challenges inherent in NGS analyses.

Ultimately, reproducibility and training are pivotal for the effective application of NGS in clinical and research settings. Our findings represent a foundational step toward addressing these issues, and we encourage education programs to adopt similar courses that assess reproducibility among bioinformatics students.

## Supporting information

S1 Text Supplementary text file. S1 Text file includes the course and project details. It also contains the details of the analyses conducted on the data.
(DOCX)

S1 Tables Supplementary file containing tables. Table A: Survey questions shared with the students. Table B: Performance metrics details. Tables C, D, and E: Precision, Recall, and F1 scores of each group’s results, respectively. Table F: Parameters examined in ANOVA analysis. Table G: Library versions in the GitHub repository and the Docker container of the Cosap tool. Table H: Genes that are contributing to the Principal Component 1.
(DOCX)

S1 Figs Supplementary file containing figures. Figs A, B, and C: Survey answers distribution. Figs D and E: Performance comparison of mappers. Fig F: Clustered heatmap of the variant lists, including the high-confidence variant list. Fig G: PCA for each pipeline configuration, grouped by the operating system and the installation method.
(DOCX)

## References

[1] Ioannidis JPA. Why most published research findings are false. PLoS Med. 2005;2(8):e124. doi: 10.1371/journal.pmed.0020124 16060722 PMC1182327

[2] Open Science Collaboration. PSYCHOLOGY. Estimating the reproducibility of psychological science. Science. 2015;349(6251):aac4716. doi: 10.1126/science.aac4716 26315443

[3] Ziemann M, Poulain P, Bora A. The five pillars of computational reproducibility: bioinformatics and beyond. Brief Bioinform. 2023;24(6):bbad375. doi: 10.1093/bib/bbad375 37870287 PMC10591307

[4] Makel MC, Plucker JA, Hegarty B. Replications in psychology research: how often do they really occur?. Perspect Psychol Sci. 2012;7(6):537–42. doi: 10.1177/1745691612460688 26168110

[5] Ioannidis J, Doucouliagos C. What’s to know about the credibility of empirical economics?. Journal of Economic Surveys. 2013;27(5):997–1004. doi: 10.1111/joes.12032

[6] Cacioppo JT, Kaplan RM, Krosnick JA, Olds JL, Dean H. Social, behavioral, and economic sciences perspectives on robust and reliable science. 1. 2015.

[7] Camerer CF, Dreber A, Holzmeister F, Ho T-H, Huber J, Johannesson M, et al. Evaluating the replicability of social science experiments in nature and science between 2010 and 2015. Nat Hum Behav. 2018;2(9):637–44. doi: 10.1038/s41562-018-0399-z 31346273

[8] Begley CG, Ellis LM. Drug development: raise standards for preclinical cancer research. Nature. 2012;483(7391):531–3. doi: 10.1038/483531a 22460880

[9] Wen H, Wang H-Y, He X, Wu C-I. On the low reproducibility of cancer studies. Natl Sci Rev. 2018;5(5):619–24. doi: 10.1093/nsr/nwy021 31258951 PMC6599599

[10] Baker M. 1,500 scientists lift the lid on reproducibility. Nature. 2016;533(7604):452–4. doi: 10.1038/533452a 27225100

[11] Jarvis MF, Williams M. Irreproducibility in preclinical biomedical research: perceptions, uncertainties, and knowledge gaps. Trends Pharmacol Sci. 2016;37(4):290–302. doi: 10.1016/j.tips.2015.12.001 26776451

[12] Errington TM, Iorns E, Gunn W, Tan FE, Lomax J, Nosek BA. An open investigation of the reproducibility of cancer biology research. Elife. 2014;3:e04333. doi: 10.7554/eLife.04333 25490932 PMC4270077

[13] Errington TM, Mathur M, Soderberg CK, Denis A, Perfito N, Iorns E, et al. Investigating the replicability of preclinical cancer biology. Elife. 2021;10:e71601. doi: 10.7554/eLife.71601 34874005 PMC8651293

[14] Goodwin S, McPherson JD, McCombie WR. Coming of age: ten years of next-generation sequencing technologies. 2016. 10.1038/nrg.2016.49 PMC10373632 27184599

[15] Qin D. Next-generation sequencing and its clinical application. Cancer Biol Med. 2019;16(1):4–10. doi: 10.20892/j.issn.2095-3941.2018.0055 31119042 PMC6528456

[16] Baykal PI, Łabaj PP, Markowetz F, Schriml LM, Stekhoven DJ, Mangul S, et al. Genomic reproducibility in the bioinformatics era. Genome Biol. 2024;25(1):213. doi: 10.1186/s13059-024-03343-2 39123217 PMC11312195

[17] Mardis ER. Next-generation DNA sequencing methods. Annu Rev Genomics Hum Genet. 2008;9:387–402. doi: 10.1146/annurev.genom.9.081307.164359 18576944

[18] Karathanasis N, Hwang D, Heng V, Abhimannyu R, Slogoff-Sevilla P, Buchel G, et al. Reproducibility efforts as a teaching tool: A pilot study. PLoS Comput Biol. 2022;18(11):e1010615. doi: 10.1371/journal.pcbi.1010615 36355750 PMC9648701

[19] Cokelaer T, Cohen-Boulakia S, Lemoine F. Reprohackathons: promoting reproducibility in bioinformatics through training. Bioinformatics. 2023;39(39 Suppl 1):i11–20. doi: 10.1093/bioinformatics/btad227 37387150 PMC10311340

[20] Firtina C, Alkan C. On genomic repeats and reproducibility. Bioinformatics. 2016;32(15):2243–7. doi: 10.1093/bioinformatics/btw139 27153582

[21] Lesack KJ, Wasmuth JD. The impact of FASTQ and alignment read order on structural variant calling from long-read sequencing data. PeerJ. 2024;12:e17101. doi: 10.7717/peerj.17101 38500526 PMC10946394

[22] Wang Q, Kotoula V, Hsu P-C, Papadopoulou K, Ho JWK, Fountzilas G, et al. Comparison of somatic variant detection algorithms using Ion Torrent targeted deep sequencing data. BMC Med Genomics. 2019;12(Suppl 9):181. doi: 10.1186/s12920-019-0636-y 31874647 PMC6929331

[23] Mercer TR, Xu J, Mason CE, Tong W, MAQC/SEQC2 Consortium. The Sequencing Quality Control 2 study: establishing community standards for sequencing in precision medicine. Genome Biol. 2021;22(1):306. doi: 10.1186/s13059-021-02528-3 34749795 PMC8574019

[24] Ergun MA, Cinal O, Bakışlı B, Emül AA, Baysan M. COSAP: comparative sequencing analysis platform. BMC Bioinformatics. 2024;25(1):130. doi: 10.1186/s12859-024-05756-z 38532317 PMC10967217

[25] Li H, Durbin R. Fast and accurate long-read alignment with Burrows-Wheeler transform. Bioinformatics. 2010;26(5):589–95. doi: 10.1093/bioinformatics/btp698 20080505 PMC2828108

[26] Langmead B, Salzberg SL. Fast gapped-read alignment with Bowtie 2. Nat Methods. 2012;9(4):357–9. doi: 10.1038/nmeth.1923 22388286 PMC3322381

[27] Cibulskis K, Lawrence MS, Carter SL, Sivachenko A, Jaffe D, Sougnez C, et al. Sensitive detection of somatic point mutations in impure and heterogeneous cancer samples. Nat Biotechnol. 2013;31(3):213–9. doi: 10.1038/nbt.2514 23396013 PMC3833702

[28] Larson DE, Harris CC, Chen K, Koboldt DC, Abbott TE, Dooling DJ, et al. SomaticSniper: identification of somatic point mutations in whole genome sequencing data. Bioinformatics. 2012;28(3):311–7. doi: 10.1093/bioinformatics/btr665 22155872 PMC3268238

[29] Kim S, Scheffler K, Halpern AL, Bekritsky MA, Noh E, Källberg M, et al. Strelka2: fast and accurate calling of germline and somatic variants. Nat Methods. 2018;15(8):591–4. doi: 10.1038/s41592-018-0051-x 30013048

[30] Fang LT, Zhu B, Zhao Y, Chen W, Yang Z, Kerrigan L, et al. Establishing community reference samples, data and call sets for benchmarking cancer mutation detection using whole-genome sequencing. Nat Biotechnol. 2021;39(9):1151–60. doi: 10.1038/s41587-021-00993-6 34504347 PMC8532138

[31] Corporation M. Windows Subsystem for Linux. 2023. https://docs.microsoft.com/en-us/windows/wsl/

[32] Institute EB. European Nucleotide Archive. https://www.ebi.ac.uk/ena/browser/home

[33] Girden ER. ANOVA: Repeated Measures. Sage. 1992.

