# Supplementary material for: From ideal to practical: Heterogeneity of student-generated variant lists highlights hidden reproducibility gaps
Source: PLoS Comput Biol. 2025 Oct 16;21(10):e1013552. doi: 10.1371/journal.pcbi.1013552 (PMC12530611; doi:10.1371/journal.pcbi.1013552)
Supplement: S1 Text — file includes the course and project details. It also contains the details of the analyses conducted on the data. (DOCX) [file pcbi.1013552.s001.docx]

**SUPPLEMENTARY MATERIAL**

# **Methods**

## **Course Content**

The course was offered as a third-year elective course in the Fall term of the 2023-2024 academic year in the Computer Engineering department at Istanbul Technical University. It spanned fourteen weeks and was structured into three main parts. The first part was concentrated on building foundational knowledge in genetics, as all students were from the computer engineering department. Over the first nine weeks, we followed selected chapters from the twelfth edition of the textbook ‘Concepts of Genetics’ by Klug et al. (2019) [1]. We conducted three practical sessions during this theoretical phase to familiarize students with the computational tools required for their projects. We designed these sessions to introduce relevant libraries, file formats, and algorithms. In the first practical session, we created a Jupyter notebook to introduce Python libraries such as Matplotlib [2], Numpy [3], and Pandas [4]. The second session was dedicated to understanding file formats like FASTQ, SAM/BAM, and VCF. Additionally, we introduced the Biopython library [5] and discussed key methods within it. The third session involved analyzing a VCF file using the cyvcf2 library [6].

After covering the genetic background and tools commonly used in bioinformatics, we set aside two weeks to discuss significant articles in the field [7,8]. Students were required to read the articles before class, and we had in-depth discussions during the sessions. The articles focused on benchmarking somatic sequencing. Through these discussions, we aimed to familiarize students with the datasets, methodologies, analyses, and visualization techniques prevalent in bioinformatics research.

In the seventh week of the course, we shared the project details with the students. The students were given two opportunities to present their results. The first round of presentations was scheduled for the twelfth week. Students presented their preliminary findings to the instructor and participated in a demonstration session with the teaching assistant. Students interpreted their results during their presentations, and the instructor provided individual feedback. In the demonstration session with the teaching assistant, students showcased how they executed their code and created visualizations for their analyses on their personal computers. We designated the thirteenth week for general feedback and revision based on the insights gained from these presentations and demonstrations. We highlighted common issues and offered suggestions for addressing them for the second round of project evaluations. We specifically focused on different visualization techniques, statistical methods, and their interpretations.

The final week was dedicated to the project evaluation’s second and final iteration. As in the previous round, students presented their findings to the instructor and participated in a demonstration session with the teaching assistant.

## **Project Details**

### *Installation*

Students were given two options for setting up the computational environment: (a) using a Docker image or (b) directly installing COSAP on their devices. The installation process using Docker required students to download the COSAP Docker image on their devices. This image includes all software requirements necessary to run COSAP. For the manual installation, students were required to install Miniconda [9], which facilitates the installation and management of necessary Python packages and NGS tools. Following this, they needed to clone the COSAP repository, install required packages, and configure environment variables.

As COSAP only supports Linux-based environments, students were advised to use either a Linux-based system or Windows Subsystem for Linux (WSL).

### *Downloading Data*

After completing the installation, students needed to download the necessary data. They downloaded paired-end FASTQ files containing tumor and normal samples' sequencing data (SRR7890850 and SRR7890851, respectively) from the SEQC2 Consortium [10], a reference genome, aligner indexes for aligning the reads, high-confidence BED files for filtering the variants for the high-confidence genomic regions, and a high-confidence variant list for performance analysis. The high-confidence reference call set was designed to serve as a ground truth benchmark. It was generated by the SEQC2 Consortium through deep whole-genome sequencing (1,500× coverage) and validated using orthogonal sequencing platforms [10].

### *Configuring and Running the Pipelines*

After completing the installations and downloading the data, students were required to modify the pipeline configuration file provided by COSAP to suit their specific needs. They were expected to run this file at least 12 times to generate the necessary VCF files. The process of executing COSAP varied depending on whether Docker or manual installation was used, necessitating the use of different commands.

### *Filtering and Analyzing the Results*

After generating VCF files, additional filtering steps were applied to the resulting variant lists. This included using BED files to focus the analysis on exomic high-confidence genomic regions and applying further filters based on the variant caller used. This ensured that the VCF files only contained variants of the highest confidence level.

The final stage of the term project involved a comprehensive analysis of pipeline outputs. Students evaluated the performance of each pipeline using metrics such as precision, recall, and F1-score. They were encouraged to use creative visualizations, including heatmaps, box-and-whisker plots, histograms, and scatter plots of principal component analysis (PCA) (with two principal components), to communicate their findings effectively. This analysis helped them understand each pipeline configuration's relative strengths and weaknesses.

### *Further Studies*

We offered an opportunity to earn bonus points for students who completed their analyses ahead of schedule. Students could gain these points by extending their analyses to include the effects of trimming, marking, or deleting duplicates. Additionally, they were encouraged to work with different FASTQ files to examine how variations in the initial input files impact the performance of the variant calling pipelines.

## **Data Analysis**

### *Principal Component Analysis*

All groups started from the same raw FASTQ reads; therefore, a fully reproducible workflow should have yielded identical variant lists for every pipeline configuration. Likewise, a list that overlaps extensively with the external high‑confidence call set would be evidence of accurate variant calling. To assess both reproducibility among student pipelines and concordance with the reference set, we analyzed all submitted variant lists using principal component analysis (PCA) [11].

Each variant list was encoded as a binary row vector. Columns corresponded to every single‑nucleotide position observed in any list, identified by the label `CHROM‑POS‑REF‑ ALT' (chromosome, position, reference allele, alternate allele). A value of 1 indicated that the variant was present in a given list; 0 indicated absence. Stacking these vectors produced a sparse binary matrix whose rows represented individual pipelines (or the high‑confidence set) and whose columns represented the union of all detected variants. Zero columns in every row were discarded to keep the matrix size tractable.

Because this matrix is both high‑dimensional and sparse, direct visual inspection is uninformative. We therefore applied PCA to project the data onto a low‑dimensional orthogonal basis that captures the greatest possible variance. The resulting component scores make it straightforward to visualise clusters of pipelines that produce similar call sets and to measure their distance from the high‑confidence reference.

PCA transforms the binary variant‑presence matrix into a set of uncorrelated, orthogonal axes (principal components) that are ranked by the amount of variation they capture across all pipelines. Concretely, the mean of each column is first subtracted so that every variant has zero mean across lists; the centred matrix X (pipelines × variants) is then factorised with a singular‑value decomposition, $X=U\Sigma V^{T}$. The right‑singular vectors $V$(eigenvectors of the sample covariance matrix) define the principal‑component directions, and the squared singular values $\sigma^{2}$give the variance explained by each component. Retaining only the leading components (here, the first two) projects every pipeline into a low‑dimensional space where Euclidean distance faithfully reflects the overall similarity of call sets. In this study, clustering near the high‑confidence benchmark in this space indicates pipelines that produce highly concordant variant lists, while greater separation flags pipelines whose results deviate from the reference or from their peers.

### *Performance Analysis*

To quantify the accuracy of each student‑generated call set, every variant list was benchmarked against the external high‑confidence catalogue. Variants present in both lists were treated as true positives, those unique to the student list as false positives, and reference variants that were missed as false negatives. Using these counts, we calculated recall (sensitivity), precision (positive‑predictive value), and their harmonic mean, the F1‑score; exact formulas are provided in Table B in S1 Tables. All metrics were computed in Python with libraries. Because multiple students implemented the same pipeline recipe (identical aligner, caller, and filtering parameters), their results were combined, forming a group for each configuration. The distributions of recall, precision, and F1 across replicates within a group were summarized with box-and-whisker plots, allowing for rapid visual comparison of typical performance and variability between pipeline configurations.

### *ANOVA Analysis*

Many sources of technical variation can influence the final set of called variants, including the chosen aligner and caller, operating system environment, package installation method, and the time each student spent on data download, alignment, and variant calling steps. To gauge the practical impact of these factors on reproducibility, we first merged all submitted variant lists with their associated metadata, including pipeline configuration, hardware and software details, and phase-specific time estimates (Table A in S1 Tables). We then summarised performance for every list by its F1‑score and used these scores as the response variable in a multifactor analysis of variance (ANOVA [12]) to identify which factors, individually or through interactions, account for a significant share of the performance variability.

ANOVA partitions the total variability of a response into components attributable to each explanatory factor and to residual (unexplained) error. For every factor, it compares the mean square between the groups defined by that factor with the mean square within those groups (i.e., the residual), forming an F‑statistic. Under the null hypothesis that group means are equal, this statistic follows an F‑distribution with degrees of freedom determined by the number of groups and the total sample size. A small p‑value, therefore, indicates that variation associated with the factor is unlikely to have arisen by chance, allowing us to conclude that the factor exerts a statistically significant effect on F1‑score.

### **References**

1. Klug WS, Cummings MR, Spencer CA, et al. Concepts of Genetics. 2019;

2. Hunter JD. Matplotlib: A 2D Graphics Environment. Computing in Science & Engineering 2007; 9:90–95

3. Harris CR, Millman KJ, van der Walt SJ, et al. Array programming with NumPy. Nature 2020; 585:357–362

4. McKinney W. Data Structures for Statistical Computing in Python. Proceedings of the 9th Python in Science Conference 2010; 56–61

5. Cock PJ, Antao T, Chang JT, et al. Biopython: freely available Python tools for computational molecular biology and bioinformatics. Bioinformatics 2009; 25:1422–1423

6. Pedersen BS, Quinlan AR. cyvcf2: fast, flexible variant analysis with Python. Bioinformatics 2017; 33:1867–1869

7. Xiao W, Ren L, Chen Z, et al. Toward best practice in cancer mutation detection with whole-genome and whole-exome sequencing. Nature biotechnology 2021; 39:1141–1150

8. Fang LT, Zhu B, Zhao Y, et al. Establishing community reference samples, data and call sets for benchmarking cancer mutation detection using whole-genome sequencing. Nature biotechnology 2021; 39:1151–1160

9. Anaconda Inc. Miniconda – A minimal installer for conda. 2021;

10. Mercer T, Xu J, Mason C, et al. The Sequencing Quality Control 2 study: establishing community standards for sequencing in precision medicine. Genome Biology 2021; 22:

11. Hotelling H. Analysis of a complex of statistical variables into principal components. Journal of Educational Psychology 1933; 24:417–441

12. Girden ER. ANOVA: Repeated measures. 1992;
