# Supplementary material for: From ideal to practical: Heterogeneity of student-generated variant lists highlights hidden reproducibility gaps
Source: PLoS Comput Biol. 2025 Oct 16;21(10):e1013552. doi: 10.1371/journal.pcbi.1013552 (PMC12530611; doi:10.1371/journal.pcbi.1013552)
Supplement: S1 Tables — Table A: Survey questions shared with the students. Table B: Performance metrics details. Tables C, D, and E: Precision, Recall, and F1 scores of each group’s results, respectively. Table F: Parameters examined in ANOVA analysis. Table G: Library versions in the GitHub repository and the Docker container of the Cosap tool. Table H: Genes that are contributing to the Principal Component 1. (DOCX) [file pcbi.1013552.s002.docx]

**SUPPLEMENTARY MATERIAL**

# **Supplementary Tables**

### Table A. Survey Questions, Answer Formats, and Response Options.

Overview of each survey question, the type of response format used, and the exact response options presented to students.

| **Q#** | **Question Text** | \| **Answer Format** \| \| --- \| | **Response Options** |
| --- | --- | --- | --- | --- |
| **1** | Which method did you use for creating your pipelines? | Multiple-choice | 1. COSAP image from Docker 2. Cloning COSAP repository from Github 3. Manually integrating aligners and variant callers (without using COSAP) |
| **2** | Which operating system did you use? | Multiple-choice | 1. Linux 2. Windows (WSL) 3. MacOS 4. Other |
| **3** | How much RAM did the machine you used have? | Multiple-choice | 1. < 8 GB 2. 8-16 GB 3. 16 GB 4. > 16 GB |
| **4** | How much disk capacity did the machine you used have? | Multiple-choice | 1. < 128 GB 2. 128-256 GB 3. 256-512 GB 4. 512 GB – 1 TB 5. > 1 TB |
| **5** | Did you purchase any additional hardware for this project? | Multiple-choice | 1. Yes 2. No |
| **6** | Was this project helpful for you to understand the new generation sequencing (NGS) technologies? | Multiple-choice | 1. Yes 2. No |
| **7** | Were the error logs provided by COSAP helpful for you? | Multiple-choice | 1. Yes 2. No |
| **8** | Was the curriculum of this course enough to understand and implement this project? | Multiple-choice | 1. Yes 2. No |
| **9** | Were explanations provided in and out of the class enough to understand the project? | Multiple-choice | 1. Yes 2. No |
| **10** | How much time did you spend on understanding the project definition? | Multiple-choice | 1. < 2 hours 2. 2-4 hours 3. 4-8 hours 4. 8-16 hours 5. > 16 hours |
| **11** | How much time did you spend on installing analysis software (COSAP or other)? | Multiple-choice | - 1. < 2 hours   2. 2-4 hours   3. 4-8 hours   4. 8-16 hours   5. > 16 hours |
| **12** | How much time did you spend on debugging the problems while downloading the necessary data? | Multiple-choice | 1. < 2 hours 2. 2-4 hours 3. 4-8 hours 4. 8-16 hours 5. > 16 hours |
| **13** | How much time did you spend on downloading the necessary data? | Multiple-choice | 1. < 2 hours 2. 2-4 hours 3. 4-8 hours 4. 8-16 hours 5. > 16 hours |
| **14** | How much time did you spend debugging the problems during the mapping phase? | Multiple-choice | 1. < 2 hours 2. 2-4 hours 3. 4-8 hours 4. 8-16 hours 5. > 16 hours |
| **15** | How much time did you spend on the mapping phase (creating BAM files)? | Multiple-choice | 1. < 2 hours 2. 2-4 hours 3. 4-8 hours 4. 8-16 hours 5. > 16 hours |
| **16** | How much time did you spend on variant calling (creating VCF files)? | Multiple-choice | 1. < 2 hours 2. 2-4 hours 3. 4-8 hours 4. 8-16 hours 5. > 16 hours |
| **17** | How much time did you spend on filtering? | Multiple-choice | 1. < 2 hours 2. 2-4 hours 3. 4-8 hours 4. 8-16 hours 5. > 16 hours |
| **18** | How much time did you spend on statistical analysis (precision, recall, f1 score)? | Multiple-choice | 1. < 2 hours 2. 2-4 hours 3. 4-8 hours 4. 8-16 hours 5. > 16 hours |
| **19** | How much time did you spend on visualization (Clustering, Heatmaps, PCA)? | Multiple-choice | 1. < 2 hours 2. 2-4 hours 3. 4-8 hours 4. 8-16 hours 5. > 16 hours |
| **20** | How much time did you spend on writing the report? | Multiple-choice | 1. < 2 hours 2. 2-4 hours 3. 4-8 hours 4. 8-16 hours 5. > 16 hours |
| **21** | How much time did you spend on preparing the presentation? | Multiple-choice | 1. < 2 hours 2. 2-4 hours 3. 4-8 hours 4. 8-16 hours 5. > 16 hours |
| **22** | Which part was the most difficult for you? | Multiple-choice | 1. Installation 2. Downloading the data 3. Mapping 4. Variant Calling 5. Filtering 6. Analysis |
| **23** | Is there anything else you want to mention? | Free-text |  |

###

### Table B. Performance metrics, formulations, and descriptions.

| **Metric** | **Formula** | **Description** |
| --- | --- | --- |
| Precision | $Precision = \frac{TP}{TP+FP}$ | Precision quantifies the accuracy of positive predictions by calculating the ratio of true positive predictions to the total number of predicted positives. TP represents the  true positives, which are correctly identified variations, while FP denotes the false positives or variants incorrectly  identified. |
| Recall | $Recall = \frac{TP}{TP + FN}$ | Recall measures the model’s ability to correctly identify all relevant positive instances. It is defined as the ratio of  true positive predictions to the total number of actual positive instances. FN represents the false negatives, which are the missed variants. |
| F1-score | $F1score =\frac{2\times Precision \times Recall}{Precision + Recall}$ | F1-Score combines both Precision and Recall into a single metric that balances their trade-off. It is defined as the harmonic mean of precision and recall. This metric ensures that both precision  and recall are considered together, with a higher F1-score indicating a better balance between the two. |

###

###

### Table C. Precision of each VCF file.

The precision values of variant lists created by each group in the study. The values are shared as percentages. A darker shade of green represents a higher value.

| **Pipeline** | **G1** | **G2** | **G3** | **G4** | **G5** | **G6** | **G7** | **G8** | **G9** | **G10** | **G11** |
| --- | --- | --- | --- | --- | --- | --- | --- | --- | --- | --- | --- |
| **Mutect-NB-BWA** | 77.75 | 80.70 | 59.27 | 22.74 | 83.92 | 79.73 | 83.95 | 79.73 | 85.09 | 57.93 | 67.66 |
| **Mutect-NB-Bowtie** | 83.30 | 89.80 | 67.81 | 25.49 | 90.13 | 84.95 | 90.23 | 84.95 | 89.70 | 14.02 | 70.08 |
| **Mutect-YB-BWA** | 86.22 | 86.49 | 63.57 | 28.12 | 84.84 | 82.50 | 86.29 | 82.50 | 81.06 | 61.76 | 70.14 |
| **Mutect-YB-Bowtie** | 87.76 | 91.33 | 70.16 | 32.23 | 91.80 | 87.21 | 87.25 | 87.21 | 87.65 | 22.02 | 71.54 |
| **SS-NB-BWA** | 30.11 | 30.11 | 9.26 | 30.11 | 30.11 | 30.11 | 30.11 | 30.11 | 30.11 | 8.92 | 8.92 |
| **SS-NB-Bowtie** | 33.42 | 33.42 | 11.72 | 33.42 | 33.42 | 33.42 | 33.42 | 33.42 | 33.42 | 11.32 | 11.72 |
| **SS-YB-BWA** | 36.92 | 36.92 | 10.30 | 37.72 | 36.92 | 36.92 | 36.92 | 36.94 | 36.94 | 9.88 | 9.88 |
| **SS-YB-Bowtie** | 44.58 | 44.58 | 14.15 | 44.58 | 44.58 | 44.58 | 44.58 | 44.58 | 44.58 | 13.64 | 14.15 |
| **Strelka-NB-BWA** | 42.79 | 42.79 | 29.76 | 0.95 | 42.79 | 42.79 | 42.81 | 42.84 | 42.84 | 28.95 | 28.30 |
| **Strelka-NB-Bowtie** | 63.29 | 63.29 | 46.53 | 1.11 | 63.29 | 63.29 | 63.29 | 63.29 | 63.29 | 0.81 | 45.19 |
| **Strelka-YB-BWA** | 48.57 | 48.57 | 33.32 | 1.23 | 48.57 | 48.60 | 48.60 | 48.65 | 48.65 | 32.46 | 31.61 |
| **Strelka-YB-Bowtie** | 70.42 | 70.42 | 53.32 | 1.53 | 70.42 | 70.42 | 70.42 | 70.42 | 70.42 | 1.06 | 51.46 |

###

### Table D. Recall of each VCF file.

The recall values of variant lists created by each group in the study. The values are shared as percentages. A darker shade of green represents a higher value.

| **Pipeline** | **G1** | **G2** | **G3** | **G4** | **G5** | **G6** | **G7** | **G8** | **G9** | **G10** | **G11** |
| --- | --- | --- | --- | --- | --- | --- | --- | --- | --- | --- | --- |
| **Mutect-NB-BWA** | 62.02 | 49.70 | 26.44 | 80.36 | 55.73 | 51.16 | 77.95 | 51.16 | 20.16 | 21.71 | 13.70 |
| **Mutect-NB-Bowtie** | 63.14 | 68.99 | 39.02 | 78.29 | 29.89 | 35.49 | 73.99 | 35.49 | 18.00 | 21.96 | 23.00 |
| **Mutect-YB-BWA** | 78.12 | 77.78 | 46.60 | 66.24 | 61.24 | 47.11 | 78.04 | 47.11 | 22.48 | 26.01 | 8.70 |
| **Mutect-YB-Bowtie** | 48.15 | 46.25 | 44.36 | 78.73 | 74.25 | 35.83 | 75.45 | 35.83 | 24.46 | 34.71 | 15.16 |
| **SS-NB-BWA** | 74.94 | 74.94 | 74.94 | 74.94 | 74.94 | 74.94 | 74.94 | 74.94 | 74.94 | 65.12 | 65.12 |
| **SS-NB-Bowtie** | 69.25 | 69.25 | 69.25 | 69.25 | 69.25 | 69.25 | 69.25 | 69.25 | 69.25 | 59.86 | 69.25 |
| **SS-YB-BWA** | 73.56 | 73.56 | 73.56 | 61.41 | 73.56 | 73.56 | 73.56 | 73.56 | 73.56 | 63.74 | 63.74 |
| **SS-YB-Bowtie** | 67.27 | 67.27 | 67.27 | 67.27 | 67.27 | 67.27 | 67.27 | 67.27 | 67.27 | 57.97 | 67.27 |
| **Strelka-NB-BWA** | 81.22 | 81.22 | 81.22 | 88.80 | 81.22 | 81.22 | 81.22 | 81.22 | 81.22 | 70.63 | 70.63 |
| **Strelka-NB-Bowtie** | 71.58 | 71.58 | 71.58 | 86.48 | 71.58 | 71.58 | 71.58 | 71.58 | 71.58 | 75.37 | 71.58 |
| **Strelka-YB-BWA** | 80.62 | 80.62 | 80.62 | 88.29 | 80.62 | 80.62 | 80.62 | 80.62 | 80.62 | 70.11 | 70.11 |
| **Strelka-YB-Bowtie** | 71.15 | 71.15 | 71.15 | 86.05 | 71.15 | 71.15 | 71.15 | 71.15 | 71.15 | 74.94 | 71.15 |

### Table E. F1-Score of each VCF file.

The F1-score values of variant lists created by each group in the study. The values are shared as percentages. A darker shade of green represents a higher value.

| **Pipeline** | **G1** | **G2** | **G3** | **G4** | **G5** | **G6** | **G7** | **G8** | **G9** | **G10** | **G11** |
| --- | --- | --- | --- | --- | --- | --- | --- | --- | --- | --- | --- |
| **Mutect-NB-BWA** | 69.00 | 61.51 | 36.57 | 35.45 | 66.98 | 62.33 | 80.84 | 62.33 | 32.59 | 31.58 | 22.78 |
| **Mutect-NB-Bowtie** | 71.83 | 78.03 | 49.54 | 38.46 | 44.89 | 50.06 | 81.31 | 50.06 | 29.99 | 17.11 | 34.63 |
| **Mutect-YB-BWA** | 81.97 | 81.90 | 53.78 | 39.48 | 71.14 | 59.98 | 81.95 | 59.98 | 35.20 | 36.61 | 15.48 |
| **Mutect-YB-Bowtie** | 62.18 | 61.41 | 54.35 | 45.73 | 82.10 | 50.79 | 80.92 | 50.79 | 38.25 | 26.95 | 25.02 |
| **SS-NB-BWA** | 42.96 | 42.96 | 16.48 | 42.96 | 42.96 | 42.96 | 42.96 | 42.96 | 42.96 | 15.68 | 15.68 |
| **SS-NB-Bowtie** | 45.08 | 45.08 | 20.05 | 45.08 | 45.08 | 45.08 | 45.08 | 45.08 | 45.08 | 19.05 | 20.05 |
| **SS-YB-BWA** | 49.17 | 49.17 | 18.07 | 46.74 | 49.17 | 49.17 | 49.17 | 49.18 | 49.18 | 17.10 | 17.10 |
| **SS-YB-Bowtie** | 53.62 | 53.62 | 23.38 | 53.62 | 53.62 | 53.62 | 53.62 | 53.62 | 53.62 | 22.08 | 23.38 |
| **Strelka-NB-BWA** | 56.05 | 56.05 | 43.56 | 1.87 | 56.05 | 56.05 | 56.06 | 56.10 | 56.10 | 41.07 | 40.40 |
| **Strelka-NB-Bowtie** | 67.18 | 67.18 | 56.40 | 2.19 | 67.18 | 67.18 | 67.18 | 67.18 | 67.18 | 1.61 | 55.40 |
| **Strelka-YB-BWA** | 60.62 | 60.62 | 47.15 | 2.43 | 60.62 | 60.64 | 60.64 | 60.68 | 60.68 | 44.37 | 43.58 |
| **Strelka-YB-Bowtie** | 70.78 | 70.78 | 60.96 | 3.01 | 70.78 | 70.78 | 70.78 | 70.78 | 70.78 | 2.10 | 59.73 |

#

###

### Table F. Parameters examined in ANOVA analysis.

The parameters analyzed in the ANOVA study.

| **No** | **Name** |
| --- | --- |
| 1 | Variant caller |
| 2 | Mapper |
| 3 | Installation method |
| 4 | Operating system |
| 5 | RAM capacity |
| 6 | Hard disk capacity |
| 7 | Survey question 7 (enough error logs) |
| 8 | Survey question 8 (enough course curriculum) |
| 9 | Survey question 10 (time for understanding project definition) |
| 10 | Survey question 11 (time for installing software) |
| 11 | Survey question 12 (time for debugging downloading) |
| 12 | Survey question 13 (time for downloading) |
| 13 | Survey question 14 (time for debugging mapping) |
| 14 | Survey question 15 (time for mapping) |
| 15 | Survey question 16 (time for variant calling) |
| 16 | Survey question 17 (time for filtering) |
| 17 | Survey question 18 (time for analysis) |
| 18 | Survey question 19 (time for visualization) |
| 19 | Survey question 20 (time for writing the report) |
| 20 | Survey question 21 (time for presentation) |
| 21 | Survey question 22 (the most difficult part) |

###

### Table G. Library versions used in Cosap’s GitHub repository and Docker image. Conda library versions used in Docker image and GitHub Repository versions of the Cosap sequencing analysis platform^^[[1]](#footnote-1)^^.

###

| **Library** | **GitHub Repository** | **Docker Image** |
| --- | --- | --- |
| python | 3.9.15 | 3.9.15 |
| bbmap | 39.01 | 39.01 |
| bowtie2 | 2.5.1 | 2.5.1 |
| bwa | 0.7.17 | 0.7.17 |
| bwa-mem2 | 2.2.1 | 2.2.1 |
| fastp | 0.23.2 | 0.23.2 |
| fastqc | 0.11.9 | 0.11.9 |
| gatk4 | 4.4.0.0 | 4.4.0.0 |
| picard | 2.27.4 | 2.27.4 |
| qualimap | 2.2.2d | 2.2.2d |
| samtools | 1.16.1 | 1.16.1 |
| somatic-sniper | 1.0.5.0 | 1.0.5.0 |
| bcftools | 1.16 | 1.16 |
| varscan | 2.4.4 | 2.4.4 |
| snakemake | 7.18.2 | 7.18.2 |
| vardict-java | 1.8.3 | 1.8.3 |
| elprep | 5.1.3 | 5.1.3 |
| snpeff | 5.1 | 5.1 |

#

### Table H. Genes that are contributing to the Principal Component 1. The top 5% of variants contributing to Principal Component 1 were compared against clinically relevant genes recommended by the American College of Medical Genetics and Genomics (ACMG)^[[2]](#footnote-2)^. Listed below are the genes in which variants were identified within Principal Component 1 (see Figure 2 in the manuscript).

| **Gene** | **Disease/Phenotype** | **Phenotype Category** |
| --- | --- | --- |
| ATP7B | Wilson disease | Other |
| BRCA1 | Hereditary breast and ovarian cancer | Cancer |
| BRCA2 | Hereditary breast and ovarian cancer | Cancer |
| HNF1A | Maturity-Onset of Diabetes of the Young | Other |
| KCNH2 | Long-QT syndrome type 2 | Cardiovascular |
| MYL2 | Hypertrophic cardiomyopathy | Cardiovascular |
| PLN | Dilated cardiomyopathy | Cardiovascular |
| PRKAG2 | Hypertrophic cardiomyopathy | Cardiovascular Metabolic |
| RB1 | Retinoblastoma | Cancer |
| TRDN | Long QT syndrome | Cardiovascular |

1. Ergün MA, Cinal O, Bakışlı B, Emül AA, Baysan M. COSAP: Comparative Sequencing Analysis Platform. BMC bioinformatics. 2024;25(1):130. [↑](#footnote-ref-1)
2. Lee K et al. ACMG SF v3.3 list for reporting of secondary findings in clinical exome and genome sequencing: A policy statement of the American College of Medical Genetics and Genomics (ACMG). Genetics in Medicine. 2025; 27(8) [↑](#footnote-ref-2)
