# Supplementary material for: From ideal to practical: Heterogeneity of student-generated variant lists highlights hidden reproducibility gaps
Source: PLoS Comput Biol. 2025 Oct 16;21(10):e1013552. doi: 10.1371/journal.pcbi.1013552 (PMC12530611; doi:10.1371/journal.pcbi.1013552)
Supplement: S1 Figs — Figs A, B, and C: Survey answers distribution. Figs D and E: Performance comparison of mappers. Fig F: Clustered heatmap of the variant lists, including the high-confidence variant list. Fig G: PCA for each pipeline configuration, grouped by the operating system and the installation method. (DOCX) [file pcbi.1013552.s003.docx]

**SUPPLEMENTARY MATERIAL**

# **Supplementary Figures**

###
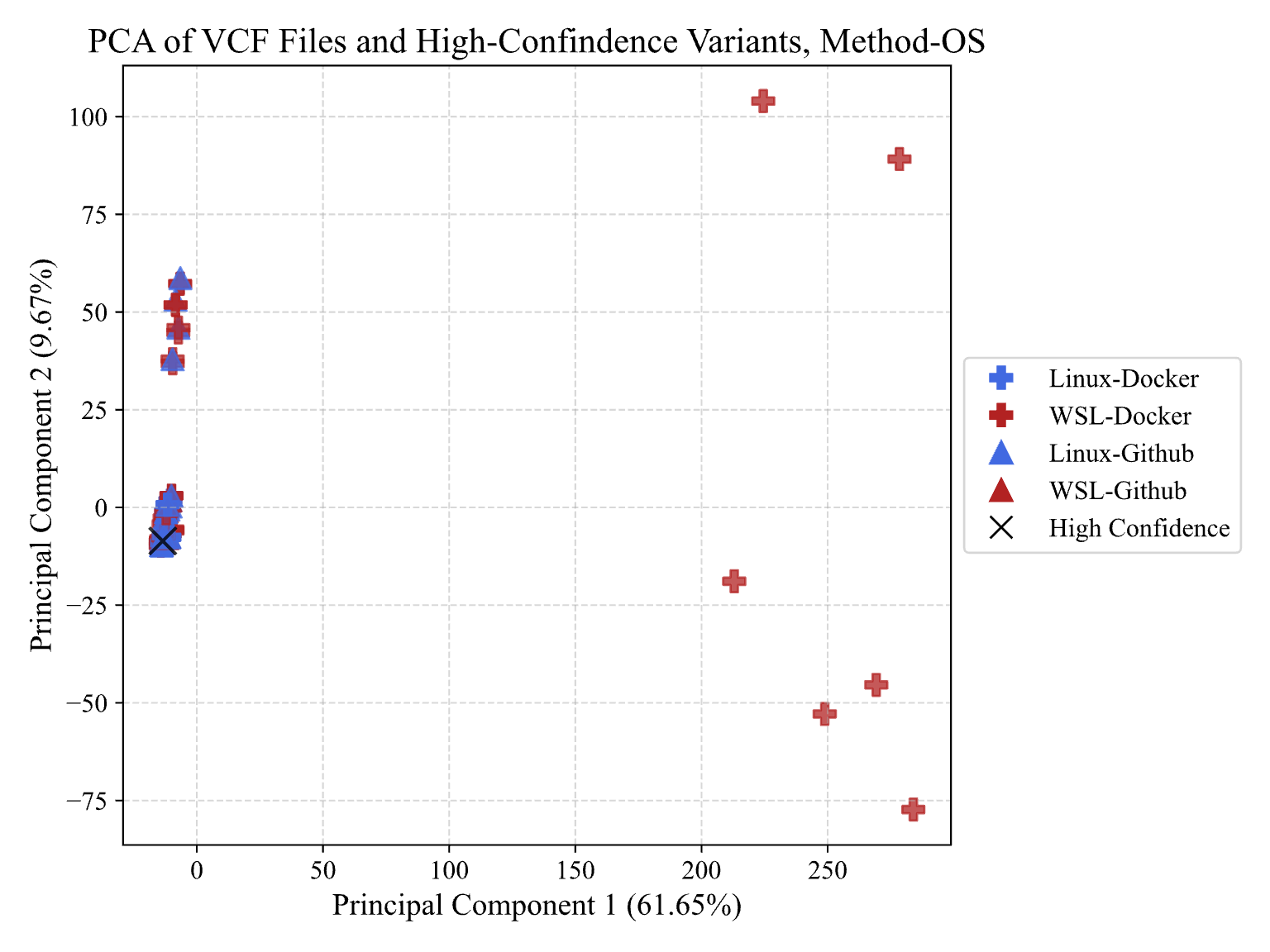


### Figure A: PCA analysis results. PCA for each pipeline configuration, along with the

### high-confidence variant list, grouped by the operating system and the installation method.


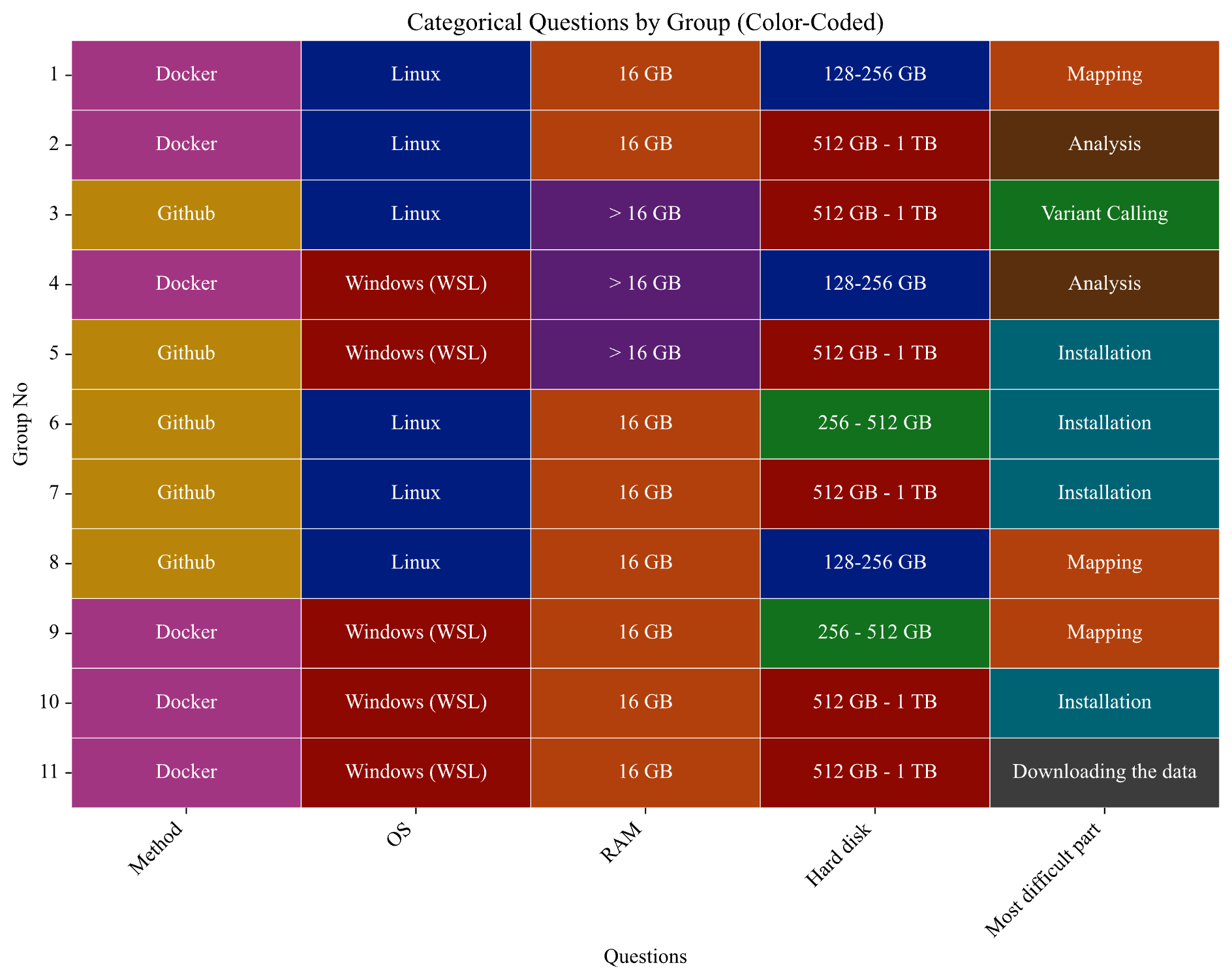


### Figure B. Survey Answers to Categorical Questions

The answers to questions 1, 2, 3, 4, and 21 in Table S1 per group.


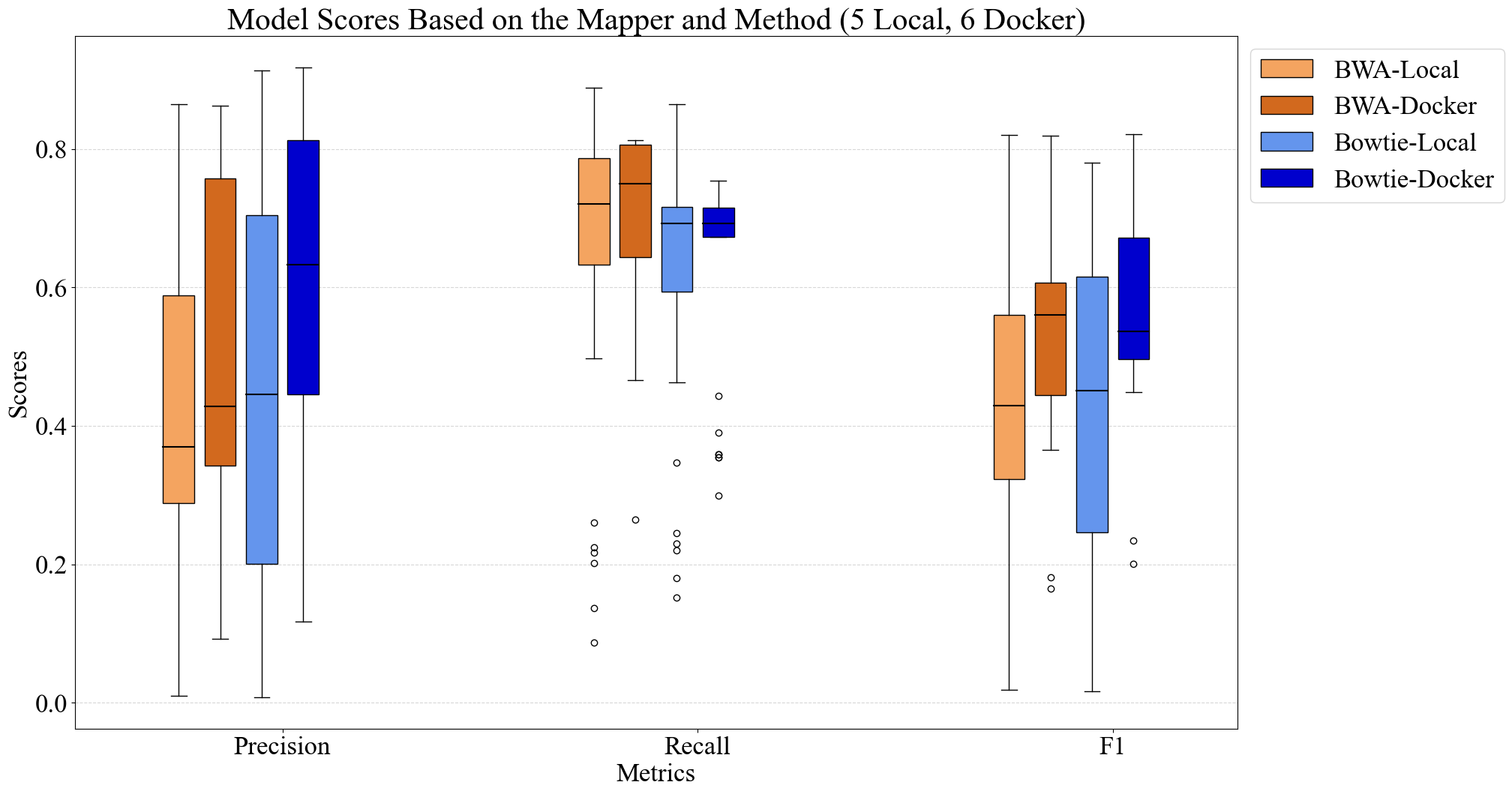


### Figure C: Performance comparison of mappers using local installation versus Docker container. The box plots display the distribution of precision, recall, and F1-scores for different aligners (BWA and Bowtie2), categorized by the installation method (local vs. Docker).


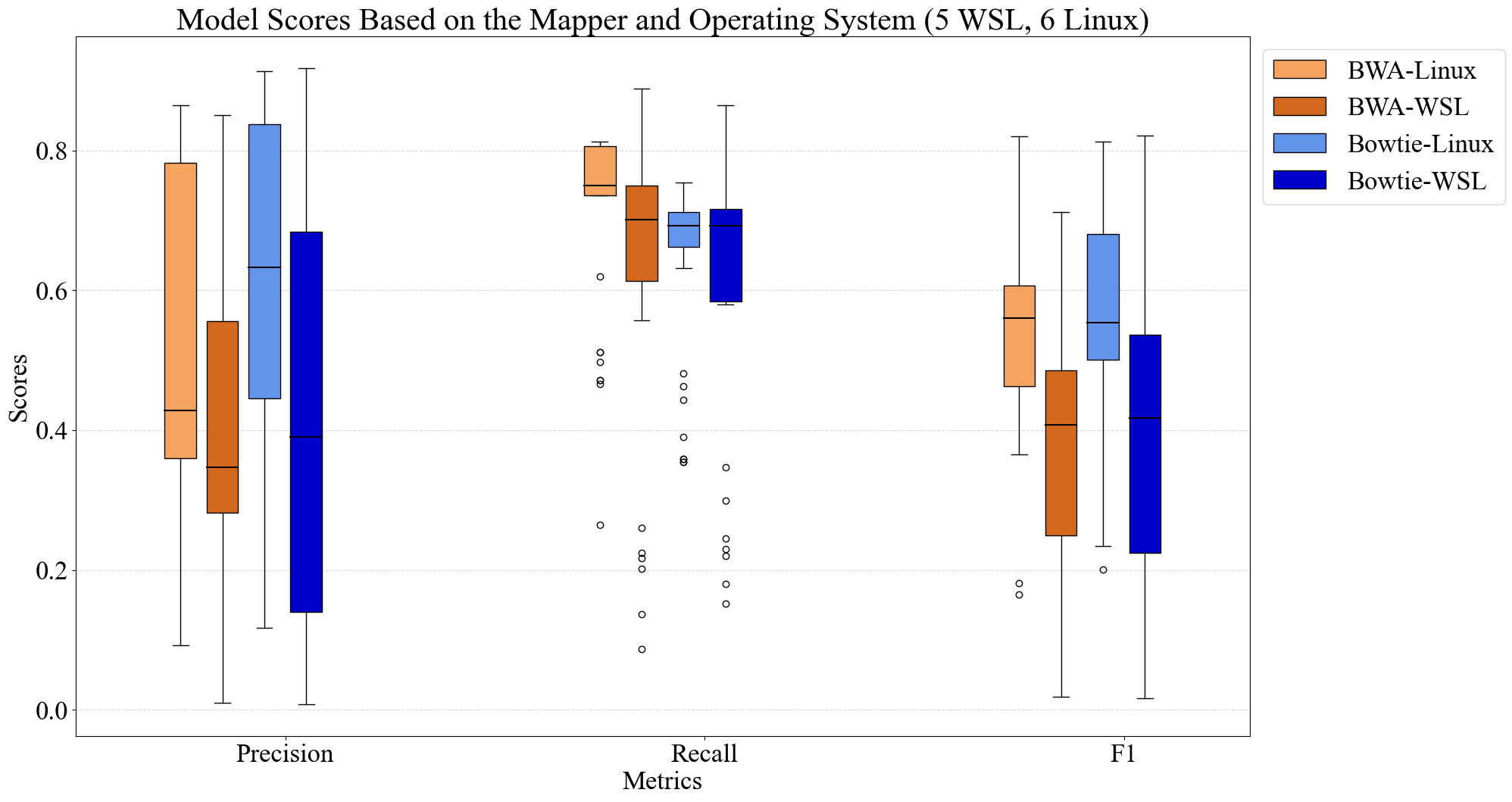


### Figure D: Performance comparison of mappers using the Linux and Windows Subsystem for Linux (WSL) operating environments. The box plots display the distribution of precision, recall, and F1-score for different aligners (BWA and Bowtie2), categorized by the operating environments (Linux vs. WSL).


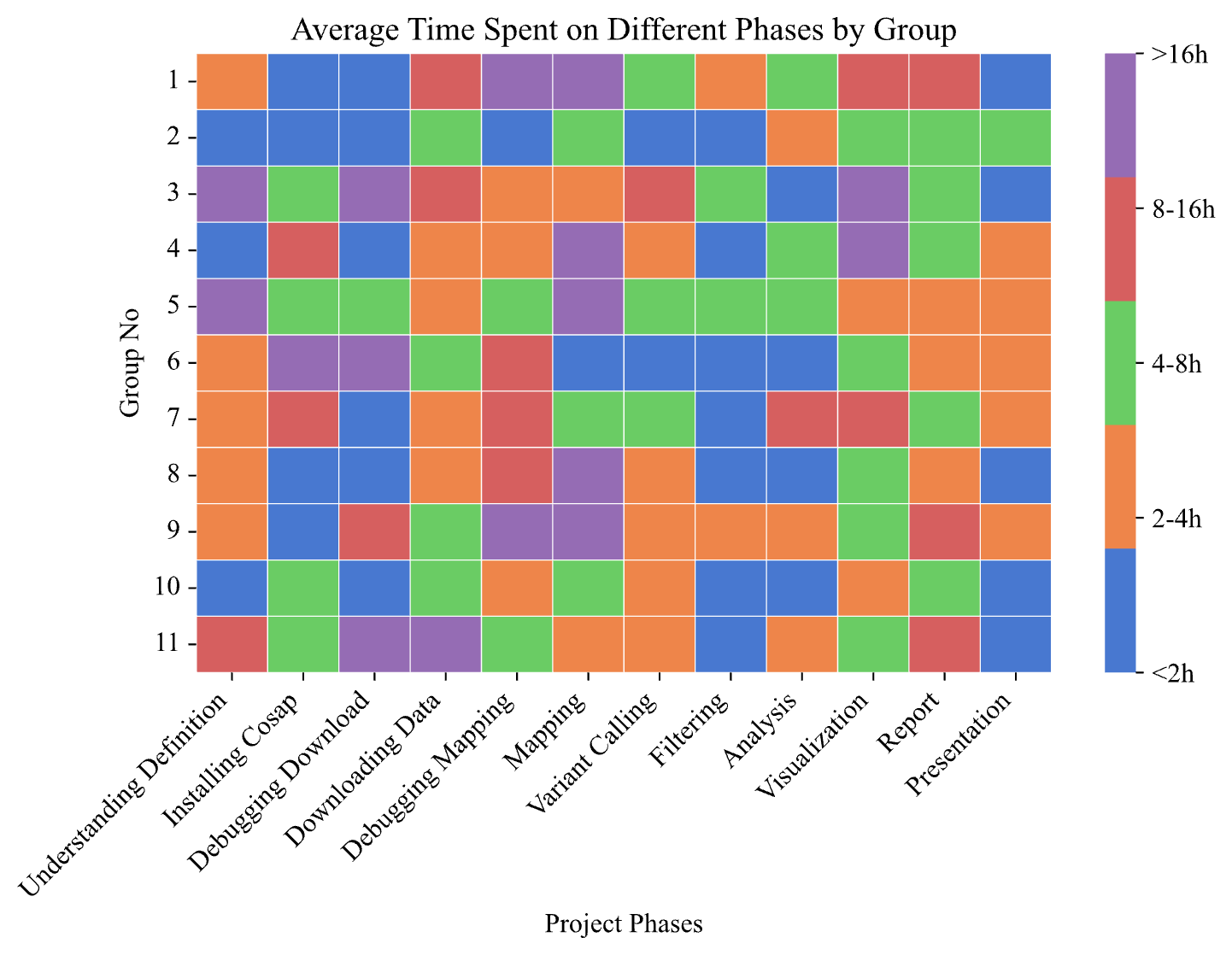


### Figure E. Survey Answers to Time Expenditure Questions

The answers to the questions between and including 10 and 20 in Table S1.


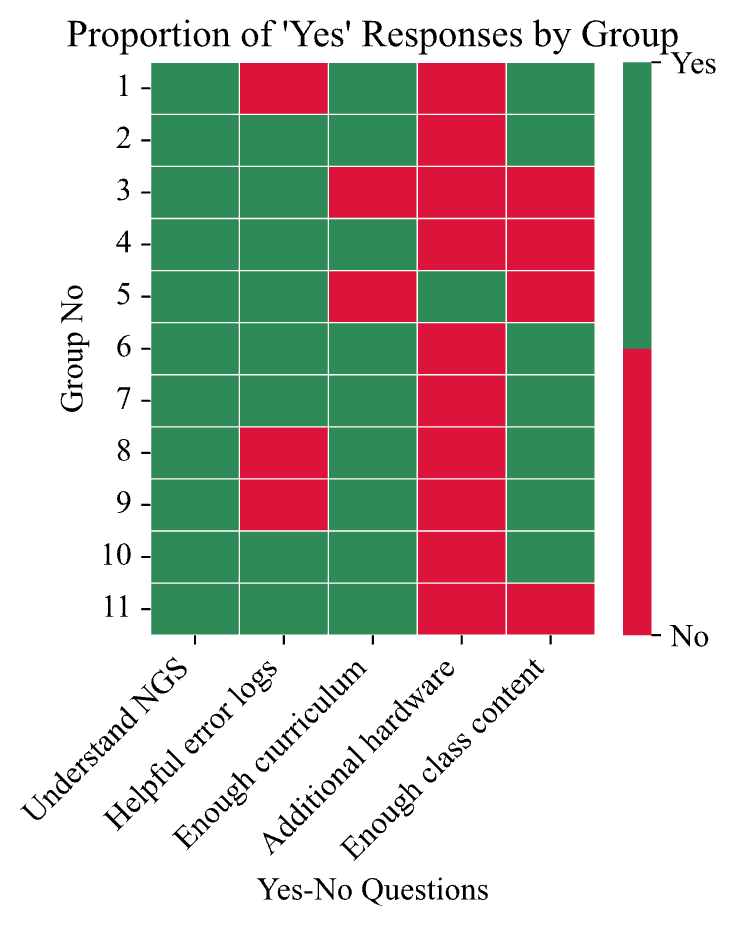


### Figure F. Survey Answers to Yes-No Questions

The answers to the questions between and including 5 and 9 in Table S1.


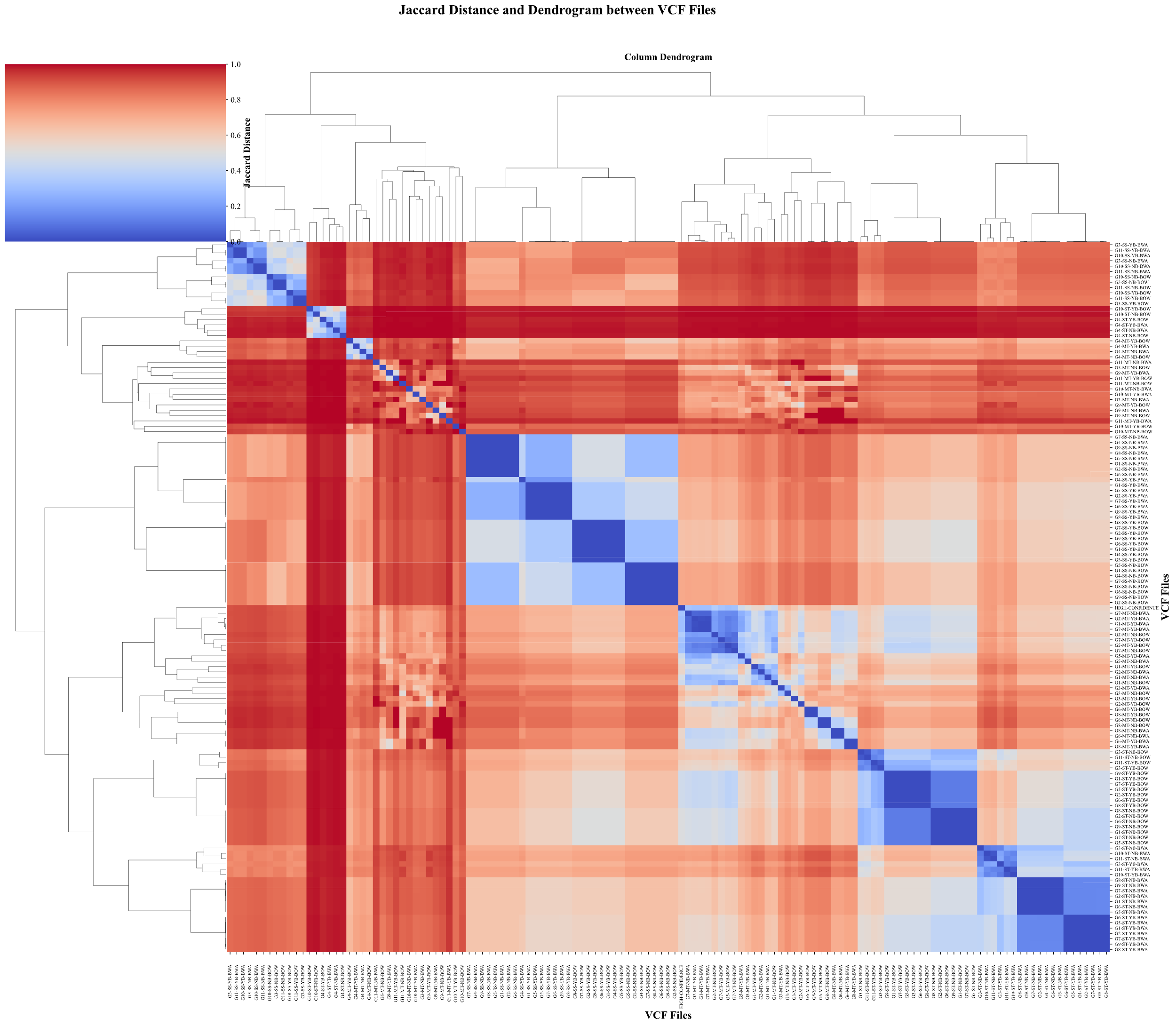


### Figure G: Clustered heatmap of Jaccard distance of all variant lists created by the student groups and the high-confidence variant list. The heatmap displays the similarity of the pipelines based on the Jaccard distance metric. Shades of blue indicate a higher rate of similarity, while shades of red show the dissimilarity of the variant lists.
